# Supplementary material for: Tead1a Initiates Transcriptional Priming Through the TEAD1a/YAP‐Notch1‐Spi1/Cebpα Axis to Promote Neutrophil Fate
Source: Adv Sci (Weinh). 2025 Aug 28;12(41):e05441. doi: 10.1002/advs.202505441 (PMC12591127; doi:10.1002/advs.202505441)
Supplement: Supplementary file 4 — Supplementary Dataset [file ADVS-12-e05441-s004.zip › 3-3.zip!/3-3/02-3.cluster/cluster6/Enrich/ego_all.pdf]

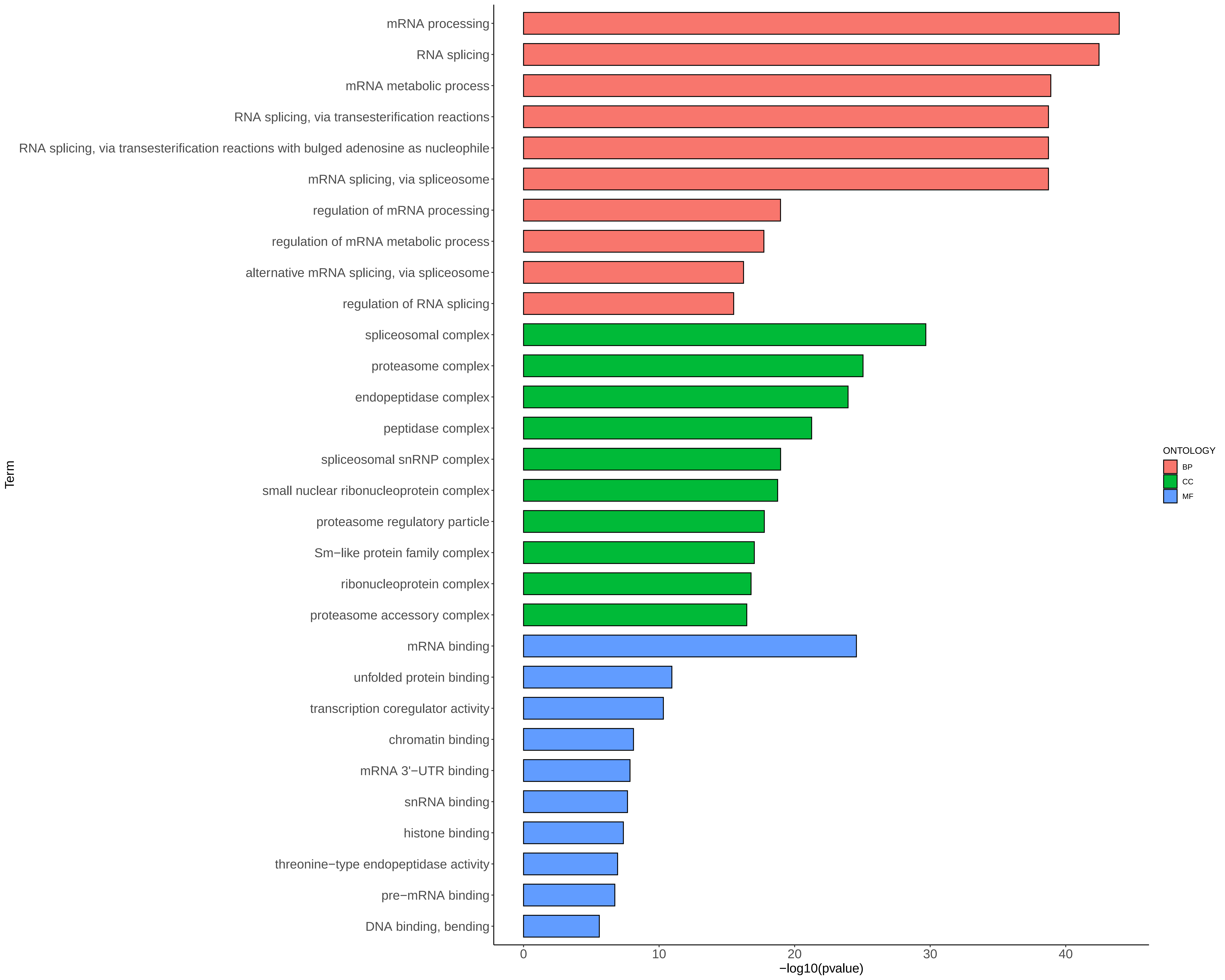
mRNA processing
RNA splicing
mRNA metabolic process
RNA splicing, via transesterification reactions
RNA splicing, via transesterification reactions with bulged adenosine as nucleophile
mRNA splicing, via spliceosome
regulation of mRNA processing
regulation of mRNA metabolic process
alternative mRNA splicing, via spliceosome
regulation of RNA splicing
spliceosomal complex
proteasome complex
endopeptidase complex
peptidase complex
spliceosomal snRNP complex
small nuclear ribonucleoprotein complex
proteasome regulatory particle
Sm−like protein family complex
ribonucleoprotein complex
proteasome accessory complex
mRNA binding
unfolded protein binding
transcription coregulator activity
chromatin binding
mRNA 3'−UTR binding
snRNA binding
histone binding
threonine−type endopeptidase activity
pre−mRNA binding
DNA binding, bending
Term
0
10
20
30
40
−log10(pvalue)
ONTOLOGY
BP
CC
MF
